# Supplementary material for: Examining the effects of transcranial direct current stimulation on human episodic memory with machine learning
Source: PLoS One. 2020 Dec 9;15(12):e0235179. doi: 10.1371/journal.pone.0235179 (PMC7725363; doi:10.1371/journal.pone.0235179)
Supplement: S1 Appendix — Russian and English words that differ significantly in the number of hits, false alarms, AUROC and reaction time. (PDF) [file pone.0235179.s001.pdf]

S1 Appendix. Significantly different words

1. Top 10 Russian words in which the number of hits in VLPFC (online encoding) and Sham groups is significantly different (higher or lower) from other words: **аудитория (auditorium), динозавр (dinosaur), дождь (rain), лектор (lecturer), паук (spider), политик (politican), почта (post), священник (priest), шея (neck), шмель (drone).**

2. Top-10 Russian words in which the number of False Alarms in VLPFC and Sham groups is significantly different (higher or lower) from other words:
   VLPFC: **балерина (ballerina), журавль (crane), тигр (tiger), почта (post), павлин (peacock), аудитория (auditorium), лектор (lecturer), пирамида (pyramid),**
   осьминог (octopus), бухгалтер (accountant). пирамида, осьминог, бухгалтер.
   Sham: **фотограф (photograph), сеть (network), змея (snake), тигр (tiger), тюлень (seal), каток (slide), шмель (drone), повар (cook), узел (knot), свинья (pig).**

3. The top-10 list of Russian English words which are differently remembered based on ROC analysis:
   Russian: **волк (wolf), кукла (doll), гитарист (guitarist), павлин (peacock), доктор (doctor), карман (pocket), туалет (toilet), психолог (psychologist), политик (politician), крыша (roof).**
   English: **athlete, politician, whiskey, skirt, drone, galaxy, infection, doctor, philosopher, astronaut.**

4. The top-10 list of Russian English words with higher Reaction Time:
   Russian: **галактика (galaxy), паломник (pilgrim), прачечная (laundry), челюсть (jaw), котенок (kitten), петиция (petition), выходной (weekend), соловей (nightingale), инфекция (infection), аптекарь (pharmacist).**
   English: **kidney, shelter, monument, snail, essay, fingerprint, petition, clown, pheasant, jumper.**

5. The top-10 list of Russian English words with lower Reaction Time:
   Russian: **пилот (pilot), сеть (network), озеро (lake), палец (finger), робот (robot), дельфин (dolphin), акула (shark), зомби (zombie), чернила (ink), праздник (party).**
   English: **prize, lotion, duck, skirt, graveyard, bubble, quiz, beach, roof, drone.**
