# Supplementary material for: Examining the effects of transcranial direct current stimulation on human episodic memory with machine learning
Source: PLoS One. 2020 Dec 9;15(12):e0235179. doi: 10.1371/journal.pone.0235179 (PMC7725363; doi:10.1371/journal.pone.0235179)
Supplement: S2 Appendix — The procedures used to augment the training set according to the method proused in [41]. (PDF) [file pone.0235179.s002.pdf]

S2 Appendix. Data Augmentations

To artificially enlarge the training set for our trials, we use the augmentation procedure proposed in the paper "Word embedding perturbation for sentence classification" by Dongxu Zhang and Zhichao Yang. We multiply each word vector by a random noise of the same shape element-wise:

$$X_{aug} = X \odot Z, \tag{1}$$

where Z has the same shape as X and is sampled from $N(\mu = 1, \sigma = 0.1)$. For the example of augmentation consult Figure 1 below.

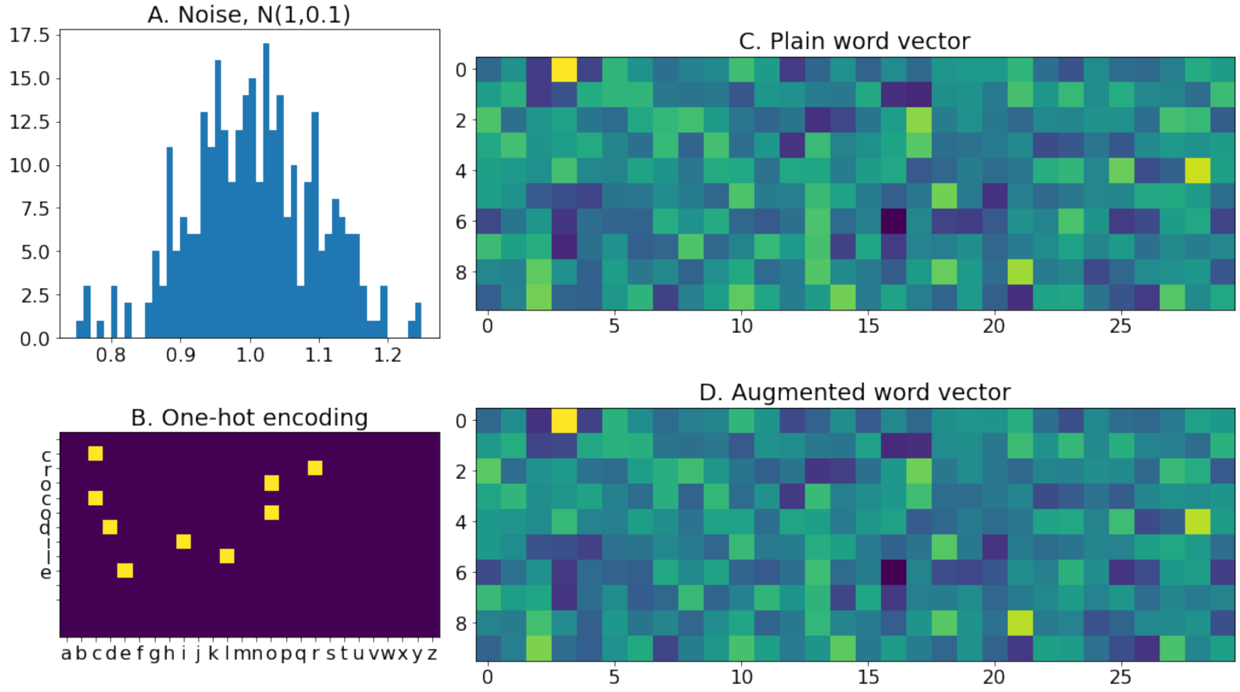

Figure 1: A. The noize we multiply our word vectors by. At B. we show the one-hot encoding of the word "crocodile". At C. you can see plain word vector and D. shows the augmented word vector for the same word, "crocodile". We reshape the vector of dimensionality 300 to a matrix of shape (10,30) to visualize. The result of augmentation is mostly the same vector with some values enhanced and some diminished.

In case of AUROC regression we also multiply the labels by a random number sampled from uniform distribution with lower bound of 0.9 and upper bound of 1.1. We run augmentation three times and combine the augmented and plain data to form the training set. The test set remains without augmentation.
