# Supplementary material for: Examining the effects of transcranial direct current stimulation on human episodic memory with machine learning
Source: PLoS One. 2020 Dec 9;15(12):e0235179. doi: 10.1371/journal.pone.0235179 (PMC7725363; doi:10.1371/journal.pone.0235179)
Supplement: S3 Appendix — The performance of participant-independent TPOT and AutoPytorch models. (PDF) [file pone.0235179.s003.pdf]

S3 Appendix. Participant-Independent ML Trials

Table 1: The performance of participant-independent TPOT models.

trial	train SCC	train SCC p-value	test SCC	test SCC p-value
Both, word vectors	0.99	0	-0.05	0.61
English word vectors	0.99	0	0.13	0.36
English, one-hot encoding	0.99	0	0.02	0.91
Russian word vectors	0.97	0	0.08	0.59
Russian word vectors	0.98	0	0.18	0.22

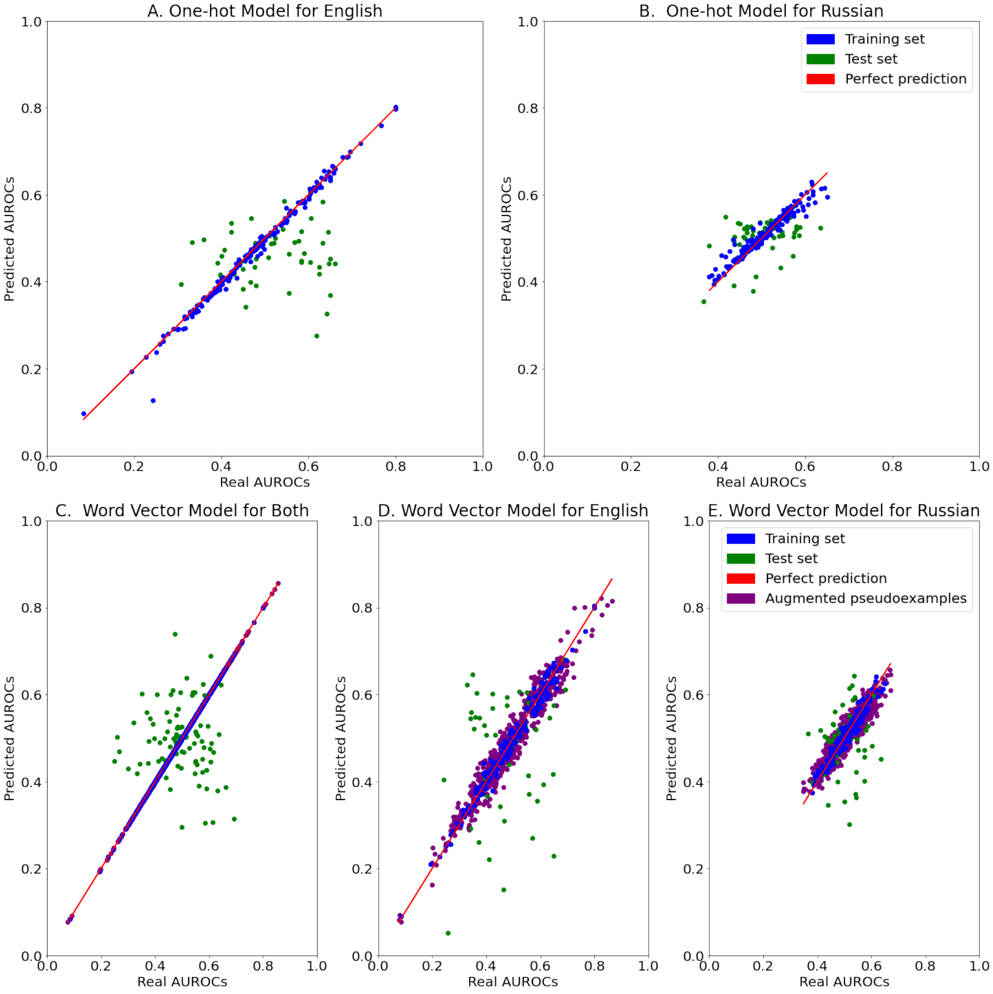

Figure 2: The prediction plots for one-hot encoding-based model for English (A) and Russian (B) words shows clear lack of generalization ability. We were able to overfit our AutoPyTorch model for the training set. Same follows for word vector-based models for both English and Russian words combined (C), only English (D) and only Russian (E).
