# Supplementary material for: Examining the effects of transcranial direct current stimulation on human episodic memory with machine learning
Source: PLoS One. 2020 Dec 9;15(12):e0235179. doi: 10.1371/journal.pone.0235179 (PMC7725363; doi:10.1371/journal.pone.0235179)
Supplement: S4 Appendix — The performance of participant-independent TPOT and AutoPytorch models. (PDF) [file pone.0235179.s004.pdf]

# S4 Appendix. Participant-Dependent ML Trials

Table 2: The performance of participant-dependent AutoPyTorch models based on one-hot encoding. Most of the times the performance is very poor both in the training and testing set. We were unable to fit a good model for each pariticpants, we didn't even get it to overfit the training set.

| trial | train AUROC ± std | test AUROC ± std |
|---|---:|---:|
| Russian All | 0.50 ± 0.01 | 0.50 ± 0.02 |
| Russian Sham | 0.50 ± 0.01 | 0.50 ± 0.02 |
| Russian VLPFC | 0.50 ± 0.00 | 0.50 ± 0.01 |
| Russian DLPFC Offline | 0.50 ± 0.02 | 0.51 ± 0.03 |
| Russian DLPFC Online | 0.50 ± 0.00 | 0.50 ± 0.02 |
| English All | 0.50 ± 0.0 | 0.50 ± 0.01 |
| English Sham | 0.50 ± 0.00 | 0.50 ± 0.01 |
| English VLPFC | 0.50 ± 0.00 | 0.50 ± 0.01 |

Table 3: The performance of participant-dependent TPOT models based on FastText word vectors. The performance is rather poor on the testing set. Unlike the models based on one-hot encoded models, we were able to overfit them on the training set with 10-fold crossvalidation, but the generalization ability remained very low.

| trial | train AUROC ± std | test AUROC ± std |
|---|---:|---:|
| Russian All | 1.0 ± 0.0 | 0.51 ± 0.07 |
| Russian Sham | 1.0 ± 0.0 | 0.52 ± 0.07 |
| Russian VLPFC | 1.0 ± 0.0 | 0.50 ± 0.07 |
| Russian DLPFC Offline | 1.0 ± 0.0 | 0.54 ± 0.08 |
| Russian DLPFC Online | 1.0 ± 0.0 | 0.51 ± 0.06 |
| English All | 1.0 ± 0.0 | 0.47 ± 0.07 |
| English Sham | 1.0 ± 0.0 | 0.47 ± 0.07 |
| English VLPFC | 1.0 ± 0.0 | 0.47 ± 0.07 |
